# DeAnnCNV: a tool for online detection and annotation of copy number variations from whole-exome sequencing data

**DOI:** 10.1093/nar/gkv556

**Published:** 2015-05-26

**Authors:** Yuanwei Zhang, Zhenhua Yu, Rongjun Ban, Huan Zhang, Furhan Iqbal, Aiwu Zhao, Ao Li, Qinghua Shi

**Affiliations:** 1 Molecular and Cell Genetics Laboratory, The CAS Key Laboratory of Innate Immunity and Chronic Disease, Hefei National Laboratory for Physical Sciences at Microscale and School of Life Sciences, University of Science and Technology of China, Hefei 230027, China; 2 School of Information Science and Technology, University of Science and Technology of China, Hefei 230027, China; 3 Institute of Pure and Applied Biology, Bahauddin Zakariya University Multan, 60800, Pakistan; 4 Hefei Institute of Physical Science, China Academy of Science, Hefei 230027, China; 5 Research Centers for Biomedical Engineering, University of Science and Technology of China, Hefei 230027, China

## Abstract

With the decrease in costs, whole-exome sequencing (WES) has become a very popular and powerful tool for the identification of genetic variants underlying human diseases. However, integrated tools to precisely detect and systematically annotate copy number variations (CNVs) from WES data are still in great demand. Here, we present an online tool, DeAnnCNV (Detection and Annotation of Copy Number Variations from WES data), to meet the current demands of WES users. Upon submitting the file generated from WES data by an in-house tool that can be downloaded from our server, DeAnnCNV can detect CNVs in each sample and extract the shared CNVs among multiple samples. DeAnnCNV also provides additional useful supporting information for the detected CNVs and associated genes to help users to find the potential candidates for further experimental study. The web server is implemented in PHP + Perl + MATLAB and is online available to all users for free at http://mcg.ustc.edu.cn/db/cnv/.

## INTRODUCTION

Various genetic variants have been associated with human diseases, of which copy number variations (CNVs) are of great importance (1) as genome-wide CNVs are reported to be involved in various human diseases including cancer (2), autism (3,4), schizophrenia (5) and intellectual disability (6). Cancer studies show that segmental deletions or duplications of chromosomes frequently occur throughout the process of tumorigenesis and progression (7,8). These aberrations are often associated with abnormal expression of tumor suppressors and oncogenes (9). Therefore, accurate detection of CNVs is an important step to identify disease-causing genes and functionally disrupted pathways.

Advances in experimental technologies from array-based technologies including array comparative genomic hybridization (10) and single nucleotide polymorphism genotyping (11) to recent high-throughput DNA sequencing (12) have greatly promoted studies on human genomes. As whole-exome sequencing (WES) continues to be cheaper and more reliable, it has been demonstrated as an effective alternative to whole-genome sequencing for the identification of genetic variants underlying human diseases.

Several state-of-the-art tools (13–16) have been developed to discover CNVs from WES data. These methods can be classified into two categories on the basis of approaches used: (i) to detect deviations in read counts among a pool of examined samples without the need of control samples, such as CoNIFER (13) and XHMM (14); (ii) to find deviations in read counts ratio by comparing the examined samples with the controls, such as ExomeCNV (15) and EXCAVATOR (16). Most of these tools are stand-alone programs that require users to locally set up computational environments with necessary hardware and software, which is sometimes difficult for users or even impossible if the technical requirements cannot be met. On the other hand, few tools are available for systematically functional annotation of CNVs by integrating currently available resources (17–20). These tools need a file containing the information of genome coordinates of CNVs as input, and annotation process is performed by finding genomic overlaps between input and annotation features. However, sample information is not provided in the annotation results from these tools, which makes it inconvenient for users to assign the annotation information to a specific sample carrying these CNVs, especially when applying these tools to annotate CNVs found in cohort studies. To our knowledge, integrated pipelines for detection and annotation of CNVs from WES data have not been reported yet. Therefore, online bioinformatics tools that can precisely detect and systematically annotate CNVs are highly needed for WES data.

Here we introduce DeAnnCNV, an efficient web server designed for integrating Detection and Annotation of Copy Number Variations from WES data. DeAnnCNV is capable of identifying CNVs from each sample accurately based on our previously published algorithm GPHMM (21) and providing detailed visualization of the detected CNVs. It can also extract CNVs shared by multiple samples and further copiously annotate them based on several supporting features including: (i) whether a CNV has been reported or not (documented in dbVar (22)); (ii) detailed information on genes associated with CNVs; (iii) whether genetic variants of these genes have been reported in human diseases (collected from ClinVar (23)); (iv) phenotypes of mice deficient for these genes (collected from Mouse Genome Informatics (MGI) (24)); (v) mRNA expression of these genes in human tissues and cell lines; (vi) functional enrichment analysis for these genes (including enriched Gene Ontology (GO), pathway and protein domains) and (vii) constructing the protein–protein interaction (PPI) network for the genes involved in CNVs, in which whether a gene is associated with a human disorder is indicated.

In order to verify the practicability of our tool, we applied DeAnnCNV to a study of infertile men and found that two patients have a CNV (each patient has only one of the two copies), which shares a gene PABPN1L, hemizygous deletion of which causes male infertility in mice. This result indicates that DeAnnCNV is a powerful and reliable tool for the detection and annotation of CNVs from WES data.

## WEB SERVER CONSTRUCTION

DeAnnCNV consists of two modules: (i) detection and visualization of CNVs from each sample, and finding CNVs shared by patients; (ii) annotation of the detected CNVs and the associated genes.

### Detection of CNVs

For both patient and control samples, read counts for all exons were extracted from each sample. In order to make read counts comparable among samples, sample normalization was performed for each patient sample by dividing the read counts of an exon by the total read counts of all exons (25). We averaged read counts from all the control samples to create a common reference that was used to represent read counts of the normal genome. The ratio between the read counts of each patient and the reference was then calculated and further normalized to eliminate Guanine-Cytosine content (GC-content) bias, which allowed ratio comparison between different genomic loci. We used the logarithm of the normalized read counts ratio to represent copy number profiles for each patient. The CNVs of each patient sample were then detected by a hidden Markov model (HMM) with hidden states corresponding to different CNVs (Supplementary Methods). Furthermore, a reliability score was calculated for each detected CNV to evaluate the reliability of DeAnnCNV results (Supplementary Methods).

In order to assess the performance of DeAnnCNV, we simulated 10 samples and each sample contained a distinct complement of 10 CNVs. Results of this simulation are presented in Supplementary Table S2. Three measurements, precision, recall and F-measure, were calculated to evaluate the CNV detection performance of DeAnnCNV (Supplementary Methods). DeAnnCNV presented high F-measure (≥0.96) across all the simulated samples, attesting the performance of DeAnnCNV for the detection of CNVs from WES data. In addition, we investigated the ability of DeAnnCNV to distinguish between different copy numbers, and the results in Supplementary Table S3 demonstrated DeAnnCNV can precisely estimate copy number for each segment. Supplementary Methods contain the detailed description of the simulation procedure and the performance evaluation strategy. Following the detection of CNVs in each sample, CNVs encompassing the overlapped genomic region were extracted as shared CNVs (for single sample, share number was set as 1).

### Annotation of CNVs

The information used for the annotation of the CNVs was collected from multiple sources and stored in MySQL. The gene location information was downloaded from Ensembl (GRCh37 and GRCh38) (26). The information on reported CNVs was downloaded from dbVar (22). The information regarding genetic variants of CNV associated genes in human disease was collected from ClinVar. The GO, pathway and protein domain information used for enrichment analysis was retrieved from DAVID (27). The enrichment *P* values were quantitatively measured by Fisher's exact test and Bonferroni correction was calculated when an adjustment was made to *P* values. The mRNA expression data were downloaded from The Human Protein Atlas (28). The PPI information was integrated from several major public databases, including HPRD (29), BioGRID (30), DIP (31), MINT (32), IntAct (33) and STRING (34) with redundant PPIs removed. The Cytoscape web application (35) was used to visualize the retrieved PPI network.

## UTILITY AND WEB INTERFACE

### Preparation of uploaded files

By using PreprocessFile package (provided by our server), files (*.count and *.gc) containing read counts for each sample and GC content of target regions can be generated from WES data and compressed into a single file (*.tar.gz) automatically. To maximize the ease for users, a shell script named ‘run.sh’ was included in the package to conveniently generate DeAnnCNV supported files.

### Data analysis

**Figure 1.**
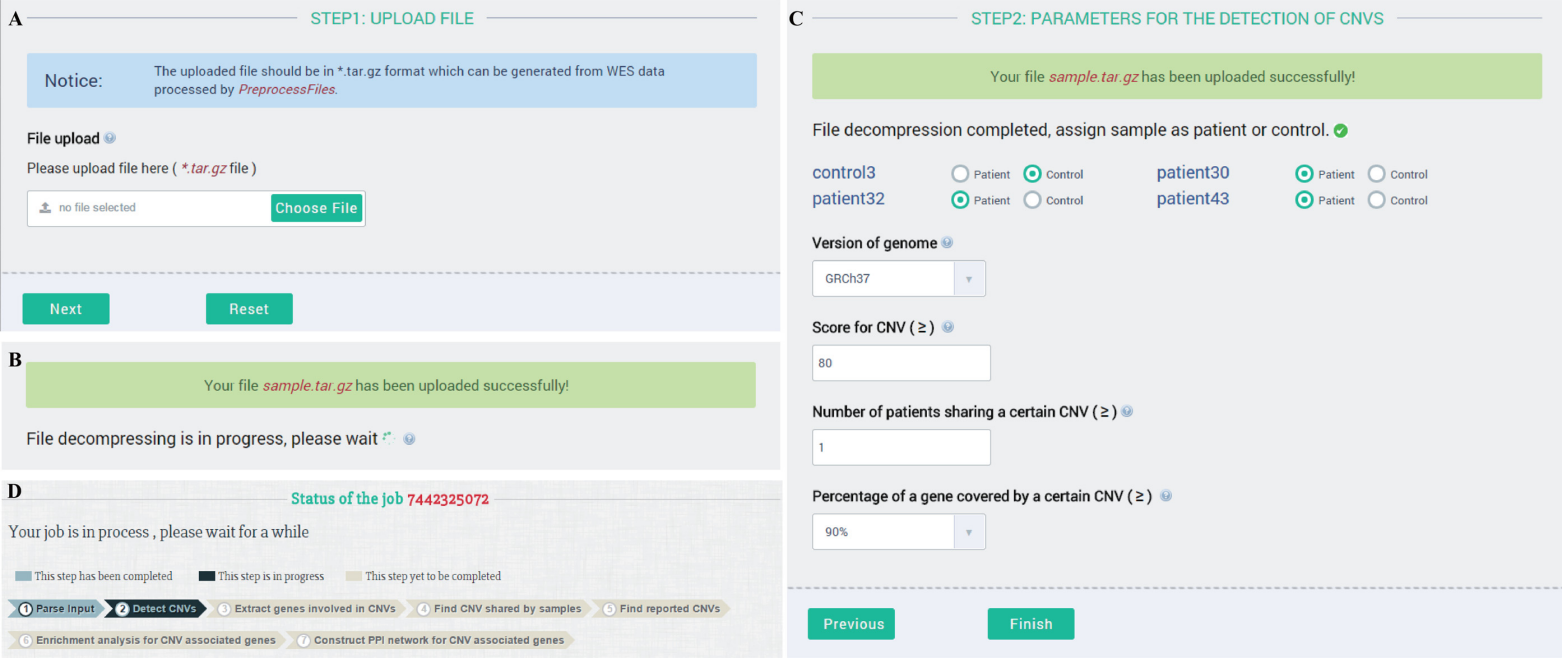
Input page of DeAnnCNV and parameters for the CNVs detection.

The compressed file (*.tar.gz) can be directly uploaded to DeAnnCNV server (Figure 1A). After the uploaded data is decompressed (Figure 1B), users will be guided to the pages where they can either choose the default parameters or set their own parameters for CNVs detection. To initiate the analysis, users should assign each sample as patient or control. If no control is assigned, DeAnnCNV will use a default control (generated from in-house WES data of normal healthy males) as the reference. Then users need to select the version of genome. Currently, our tool supports GRCh37 and GRCh38. Following this, users have an option to set the threshold score (default 80) which descripts the strength of the evidence for the detected CNVs. In addition, users can modify the parameter ‘Number of patients sharing the same CNV’ to fetch shared CNVs among multiple samples (for single sample, share number set as 1). Users can also optimize the percentage of a gene covered by a CNV while defining CNV associated genes. Once all the parameters are set, users should click the ‘Finish’ button to start analysis (Figure 1C). A page will be provided to display the status of a job and link to the detailed results (Figure 1D).

### Results and description

Once a job is completed, the results page will display the parameters for the detection of CNVs and provide links to the detailed results. These results include CNVs associated information, enrichment analysis and PPI network.

#### CNV associated information

**Figure 2.**
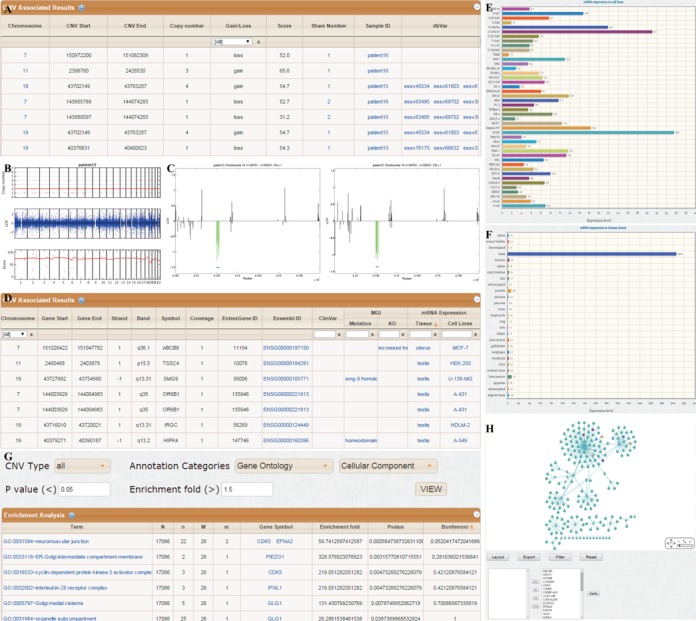
An example output page of DeAnnCNV.

This page will display the detailed information on CNVs and associated genes. For CNVs, the information includes: the chromosome location of each CNV, copy number, copy number gain (copy number > 2) or loss (copy number < 2), score of the CNV, sample carrying the CNV, number of samples sharing the same CNVs and whether these CNVs are reported in dbVar (Figure 2A). By clicking the sample ID or share number, figures illustrating all the CNVs detected in this sample or all the samples carrying a specific CNV will be displayed, respectively (Figure 2B and C). For genes involved in the CNVs, the information includes: chromosome location of genes, percentage of genes covered by a certain CNV (coverage), association with human disease reported in ClinVar, phenotypes of deficient mice in MGI and the mRNA expression in human tissues and cell lines is provided (Figure 2D). By clicking the names of tissues or cell lines, corresponding histogram will be displayed to illustrate the mRNA expression (Figure 2E and F).

#### Enrichment analysis

This page will display the enrichment analysis results for CNVs associated genes including: the enriched chromosomes, GO terms, pathways and protein domains. The enrichment fold, *P* values (Fisher's exact test) and Bonferroni adjusted *P* values are displayed for each annotated term (Figure 2G). Users can select CNV type and annotation categories from the drop-down box and optimize *P* value and enrichment fold to refine the results. The detailed information for each enriched term can be viewed by clicking the term and a page will display the resource providing this annotation information. Users can save results of enrichment analysis by clicking download button in the footer navigation bar of Enrichment analysis page.

#### PPI network

This page will display the PPI network for the genes involved in the detected CNVs to determine if some already reported disease-causing genes interact with them. CNV associated genes will be presented in diamonds and their interacted genes will be presented in circles. Nodes with color represent genes with disease information reported in ClinVar. By clicking the colored node, the information of disease will be displayed (Figure 2H).

## CASE STUDY AND DISCUSSION

**Figure 3.**
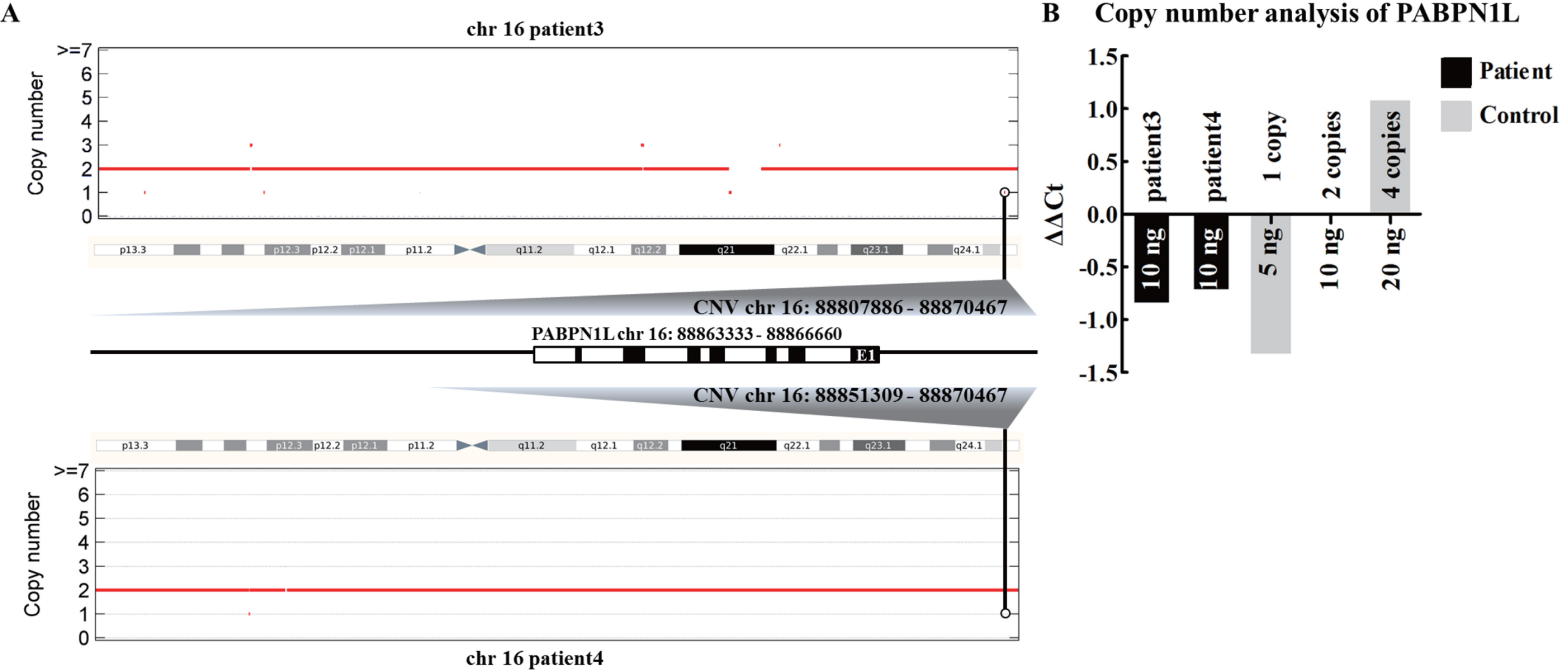
Candidate CNVs detected by DeAnnCNV were confirmed by qPCR. Schematic representation of CNVs in genomic region (**A**). Control samples with different gDNA amounts (5, 10 and 20 ng) were used to imitate the ΔCT generated by one copy, two copies and four copies of PABPN1L. Ten nanogram of patients gDNA was used as templates. The ΔΔCT value for 10 ng of control gDNA was set as 0. All samples were then normalized to the calibrator to determine ΔΔCT values (**B**).

We have performed WES for male infertile patients with the same clinical phenotype, however, no potential disease-causing candidate variants (both SNVs and Indels) were found in exome region. Thus, we tried to explore whether there are some potential CNVs related to male infertility. After uploading the files generated from WES data of four patients to DeAnnCNV server, a total of 45 CNVs (score > 80) and 256 associated genes (coverage > 90%) were detected in these patients. The detailed results can be accessed at http://mcg.ustc.edu.cn/db/cnv/check.php?rand_num=7235007981. Through sorting in the ‘gain/loss’ column of the results table, 13 CNVs with copy number loss were selected for further analysis (Supplementary Figure S1A). From the phenotype information presented by DeAnnCNV, we focused on the CNVs that encompassed the genes that contain reported infertility-associated variations in ClinVar or the genes for which the deficient mice are infertile or subfertile. Interestingly, by searching for ‘infertility’ in the ‘MGI-KO’ column, we found that two patients, patients 3 and 4, carried two CNVs (copy number = 1) that shared a gene PABPN1L, localized at human 16q24.3. According to MGI annotation, male mice with hemizygous deletion of *Pabpn1l* (localized at mouse chromosome 8) are infertile (Supplementary Figure S1B). Interestingly, the carrying of the two CNVs in the patients was confirmed experimentally by using the ΔΔCT-method as previously described (36,37). A reduction of PABPN1L gDNA amount was observed in both patients compared with control (Figure 3). Consistently, in the ‘mRNA Expression’ column of the results table, PABPN1L was found to express in human testis (Supplementary Figure S1B). Besides, in patients 3 and 4, we did not detect any other CNVs that encompassed genes that highly expressed in human testis or damaged human and mouse fertility after mutated. Taken together, these results indicated that loss of a copy of PABPN1L is the most probable cause of male infertility in these two patients, although functional research on how haploinsufficiency in PABPN1L results in male infertility is needed. This case study indicates that DeAnnCNV can not only detect CNVs based on WES data but also help the users to find out potential disease-associated CNVs by comprehensive annotation of detected CNVs and associated genes.

## CONCLUSION

In conclusion, we have described the DeAnnCNV web server, a web-based tool that is capable of systematic detection and annotation of CNVs from WES data. This server has integrated two separated modules, detection and annotation of CNVs. With the annotation of genes involved in CNVs, users can conveniently screen the potential disease-associated CNVs.

## SUPPLEMENTARY DATA

Supplementary Data are available at NAR Online.

SUPPLEMENTARY DATA

## References

[1] Sharp A.J., Locke D.P., McGrath S.D., Cheng Z., Bailey J.A., Vallente R.U., Pertz L.M., Clark R.A., Schwartz S., Segraves R. (2005). Segmental duplications and copy-number variation in the human genome. Am. J. Hum. Genet..

[2] Yu Z., Liu Y., Shen Y., Wang M., Li A. (2014). CLImAT: accurate detection of copy number alteration and loss of heterozygosity in impure and aneuploid tumor samples using whole-genome sequencing data. Bioinformatics.

[3] Sebat J., Lakshmi B., Malhotra D., Troge J., Lese-Martin C., Walsh T., Yamrom B., Yoon S., Krasnitz A., Kendall J. (2007). Strong association of de novo copy number mutations with autism. Science.

[4] Glessner J.T., Wang K., Cai G., Korvatska O., Kim C.E., Wood S., Zhang H., Estes A., Brune C.W., Bradfield J.P. (2009). Autism genome-wide copy number variation reveals ubiquitin and neuronal genes. Nature.

[5] Clair D. (2009). Copy number variation and schizophrenia. Schizophr. Bull..

[6] Madrigal I., Rodriguez-Revenga L., Armengol L., Gonzalez E., Rodriguez B., Badenas C., Sanchez A., Martinez F., Guitart M., Fernandez I. (2007). X-chromosome tiling path array detection of copy number variants in patients with chromosome X-linked mental retardation. BMC Genomics.

[7] Albertson D.G., Collins C., McCormick F., Gray J.W. (2003). Chromosome aberrations in solid tumors. Nat. Genet..

[8] Stratton M.R., Campbell P.J., Futreal P.A. (2009). The cancer genome. Nature.

[9] Bentires-Alj M., Gil S.G., Chan R., Wang Z.C., Wang Y., Imanaka N., Harris L.N., Richardson A., Neel B.G., Gu H. (2006). A role for the scaffolding adapter GAB2 in breast cancer. Nat. Med..

[10] Park P.J. (2008). Experimental design and data analysis for array comparative genomic hybridization. Cancer Invest..

[11] Li A., Liu Y., Zhao Q., Feng H., Harris L., Wang M. (2014). Genome-wide identification of somatic aberrations from paired normal-tumor samples. PloS One.

[12] Schuster S.C. (2008). Next-generation sequencing transforms today's biology. Nat. Methods.

[13] Krumm N., Sudmant P.H., Ko A., O'Roak B.J., Malig M., Coe B.P., Quinlan A.R., Nickerson D.A., Eichler E.E. (2012). Copy number variation detection and genotyping from exome sequence data. Genome Res..

[14] Fromer M., Moran J.L., Chambert K., Banks E., Bergen S.E., Ruderfer D.M., Handsaker R.E., McCarroll S.A., O'Donovan M.C., Owen M.J. (2012). Discovery and statistical genotyping of copy-number variation from whole-exome sequencing depth. Am. J. Hum. Genet..

[15] Sathirapongsasuti J.F., Lee H., Horst B.A., Brunner G., Cochran A.J., Binder S., Quackenbush J., Nelson S.F. (2011). Exome sequencing-based copy-number variation and loss of heterozygosity detection: ExomeCNV. Bioinformatics.

[16] Magi A., Tattini L., Cifola I., D'Aurizio R., Benelli M., Mangano E., Battaglia C., Bonora E., Kurg A., Seri M. (2013). EXCAVATOR: detecting copy number variants from whole-exome sequencing data. Genome Biol..

[17] Erikson G.A., Deshpande N., Kesavan B.G., Torkamani A. (2014). SG-ADVISER CNV: copy-number variant annotation and interpretation.

[18] Zhao M., Zhao Z. (2013). CNVannotator: a comprehensive annotation server for copy number variation in the human genome. PloS One.

[19] Chang X., Wang K. (2012). wANNOVAR: annotating genetic variants for personal genomes via the web. J. Med. Genet..

[20] Wang K., Li M., Hadley D., Liu R., Glessner J., Grant S.F., Hakonarson H., Bucan M. (2007). PennCNV: an integrated hidden Markov model designed for high-resolution copy number variation detection in whole-genome SNP genotyping data. Genome Res..

[21] Li A., Liu Z., Lezon-Geyda K., Sarkar S., Lannin D., Schulz V., Krop I., Winer E., Harris L., Tuck D. (2011). GPHMM: an integrated hidden Markov model for identification of copy number alteration and loss of heterozygosity in complex tumor samples using whole genome SNP arrays. Nucleic Acids Res..

[22] Lappalainen I., Lopez J., Skipper L., Hefferon T., Spalding J.D., Garner J., Chen C., Maguire M., Corbett M., Zhou G. (2013). DbVar and DGVa: public archives for genomic structural variation. Nucleic Acids Res..

[23] Landrum M.J., Lee J.M., Riley G.R., Jang W., Rubinstein W.S., Church D.M., Maglott D.R. (2014). ClinVar: public archive of relationships among sequence variation and human phenotype. Nucleic Acids Res..

[24] Blake J.A., Bult C.J., Eppig J.T., Kadin J.A., Richardson J.E. (2014). The Mouse Genome Database: integration of and access to knowledge about the laboratory mouse. Nucleic Acids Res..

[25] Klambauer G., Schwarzbauer K., Mayr A., Clevert D.A., Mitterecker A., Bodenhofer U., Hochreiter S. (2012). cn.MOPS: mixture of Poissons for discovering copy number variations in next-generation sequencing data with a low false discovery rate. Nucleic Acids Res..

[26] Cunningham F., Amode M.R., Barrell D., Beal K., Billis K., Brent S., Carvalho-Silva D., Clapham P., Coates G., Fitzgerald S. (2015). Ensembl 2015. Nucleic Acids Res..

[27] Huang da W., Sherman B.T., Lempicki R.A. (2009). Bioinformatics enrichment tools: paths toward the comprehensive functional analysis of large gene lists. Nucleic Acids Res..

[28] Uhlen M., Fagerberg L., Hallstrom B.M., Lindskog C., Oksvold P., Mardinoglu A., Sivertsson A., Kampf C., Sjostedt E., Asplund A. (2015). Proteomics. Tissue-based map of the human proteome. Science.

[29] Keshava Prasad T.S., Goel R., Kandasamy K., Keerthikumar S., Kumar S., Mathivanan S., Telikicherla D., Raju R., Shafreen B., Venugopal A. (2009). Human Protein Reference Database–2009 update. Nucleic Acids Res..

[30] Chatr-Aryamontri A., Breitkreutz B.J., Oughtred R., Boucher L., Heinicke S., Chen D., Stark C., Breitkreutz A., Kolas N., O'Donnell L. (2015). The BioGRID interaction database: 2015 update. Nucleic Acids Res..

[31] Salwinski L., Miller C.S., Smith A.J., Pettit F.K., Bowie J.U., Eisenberg D. (2004). The Database of Interacting Proteins: 2004 update. Nucleic Acids Res..

[32] Licata L., Briganti L., Peluso D., Perfetto L., Iannuccelli M., Galeota E., Sacco F., Palma A., Nardozza A.P., Santonico E. (2012). MINT, the molecular interaction database: 2012 update. Nucleic Acids Res..

[33] Orchard S., Ammari M., Aranda B., Breuza L., Briganti L., Broackes-Carter F., Campbell N.H., Chavali G., Chen C., del-Toro N. (2014). The MIntAct project–IntAct as a common curation platform for 11 molecular interaction databases. Nucleic Acids Res..

[34] Franceschini A., Szklarczyk D., Frankild S., Kuhn M., Simonovic M., Roth A., Lin J., Minguez P., Bork P., von Mering C. (2013). STRING v9.1: protein-protein interaction networks, with increased coverage and integration. Nucleic Acids Res..

[35] Shannon P., Markiel A., Ozier O., Baliga N.S., Wang J.T., Ramage D., Amin N., Schwikowski B., Ideker T. (2003). Cytoscape: a software environment for integrated models of biomolecular interaction networks. Genome Res..

[36] Li Y., Pabst S., Lokhande S., Grohe C., Wollnik B. (2009). Extended genetic analysis of BTNL2 in sarcoidosis. Tissue Antigens.

[37] Beleggia F., Li Y., Fan J., Elcioglu N.H., Toker E., Wieland T., Maumenee I.H., Akarsu N.A., Meitinger T., Strom T.M. (2015). CRIM1 haploinsufficiency causes defects in eye development in human and mouse.

