# Supplementary material for: DeAnnCNV: a tool for online detection and annotation of copy number variations from whole-exome sequencing data
Source: Nucleic Acids Res. 2015 May 26;43(Web Server issue):W289–94. doi: 10.1093/nar/gkv556 (PMC4489280; doi:10.1093/nar/gkv556)
Supplement: SUPPLEMENTARY DATA [file supp_43_W1_W289__index.html]

DeAnnCNV: a tool for online detection and annotation of copy number variations from whole-exome sequencing data — SUPPLEMENTARY DATA 

# DeAnnCNV: a tool for online detection and annotation of copy number variations from whole-exome sequencing data

## SUPPLEMENTARY DATA

- SUPPLEMENTARY DATA
