# Supplementary material for: DeAnnCNV: a tool for online detection and annotation of copy number variations from whole-exome sequencing data
Source: Nucleic Acids Res. 2015 May 26;43(Web Server issue):W289–94. doi: 10.1093/nar/gkv556 (PMC4489280; doi:10.1093/nar/gkv556)
Supplement: SUPPLEMENTARY DATA [file supp_gkv556_nar-00361-web-b-2015-File005.pdf]

Supplementary Material

DeAnnCNV: a tool for online detection and annotation of copy number variations from whole-exome sequencing data

Yuanwei Zhang[1*], Zhenhua Yu[2*], Rongjun Ban[2*], Huan Zhang[1*], Furhan Iqbal[1, 3], Ao Li[2, 4†], and Qinghua Shi[1†]

[1]Molecular and Cell Genetics Laboratory, The CAS Key Laboratory of Innate Immunity and Chronic Disease, Hefei National Laboratory for Physical Sciences at Microscale and School of Life Sciences, University of Science and Technology of China , Hefei 230027, China.[2]School of Information Science and Technology, University of Science and Technology of China, Hefei 230027, China.[3]Institute of Pure and Applied Biology, Bahauddin Zakariya University Multan, 60800, Pakistan.[4]Research Centers for Biomedical Engineering, University of Science and Technology of China, Hefei 230027, China.

*These authors contributed equally to this manuscript.

†To whom correspondence should be addressed.

1. Supplementary Methods

Hidden Markov model

The hidden states of the HMM is depicted in Table S1. Each hidden state corresponds to one type of the CNVs ranging from 0 to 7 copies. Copy number of each exon is represented by the LCR of the exon as defined in the main text. We assume that LCR is Student's t-distributed with the emission probability under each hidden state defined as:

$$p(l_i \mid c, \sigma, \nu, o) = \frac{\Gamma((\nu+1)/2)}{\Gamma(\nu/2)\sqrt{\pi\nu}\sigma}(1+\frac{1}{\nu}(\frac{l_i-\mu_c}{\sigma})^2)^{-\frac{\nu+1}{2}} \quad (1)$$

where ν is the number of degrees of freedom and Γ is the gamma function. μ_c is the mean value of the LCR signals under hidden state *c* and defined as:

$$\mu_c = \log 2(y_c / 2) + o \quad (2)$$

Parameter o is introduced to account for possible baseline shift of the LCR signals, and y_c denotes the copy number associated with hidden state *c*.

We adopted expectation maximization (EM) algorithm (1) to learn the HMM and estimate model parameters. In the expectation step of the EM algorithm, expectation of the partial log-likelihood function of LCR signals was formulated as:

$$E(LL_l = \sum_{i=1}^{N}\sum_{c=1}^{C}\gamma_{i,c}\log(\,p(l_i \mid c,\sigma,\nu,o))) \tag{4}$$

Forward-backward algorithm (2) was employed to calculate the posterior probability $\gamma_{i,c}$ that the *i*th exon is in hidden state *c*. In the maximization step, all parameters were updated by using Newton–Raphson method. The parameter updating procedure was performed iteratively until the EM algorithm converges. Once the training procedure was finished, copy number of each exon was inferred from the hidden state associated with the largest posterior probability. At the same time, segmentation of all exons based on the copy numbers was performed to output CNVs for each sample.

Reliability score

It is necessary to provide a measurement for users to evaluate the reliability of DeAnnCNV results. Based on the segmentation results, we defined a reliability score for each segment as follows:

$$Score_i = mean\left(\frac{p(l_{ij} \mid c,\sigma,\nu,o)}{p(\hat{l}_{ij} \mid c,\sigma,\nu,o)}\right) \tag{5}$$

where l_{ij} is the LCR value of the *j*th exon in the *i*th segment and $\hat{l}_{ij}$ is the expected LCR value in state *c*. Furthermore, the scores for all segments along the whole genome were scaled to 0~100.

Simulated datasets

We simulated ten samples to examine the CNV detection performance of DeAnnCNV. Sequencing data from a real normal sample was used to generate the simulated samples with each sample containing a distinct complement of CNVs as illustrated in Table S4-Table S13. The CNVs presented in each sample range from one to twenty copies, and the size ranges from 500kb to 4.5Mb. We generated the sequencing data of each simulated sample by following two steps: 1) for a given region with copy number of *C*, reads mapped to the

region were randomly and repeatedly sampled from the real normal BAF file, the total number of reads sampled from the region is $N \cdot C/2$, where N is number of reads aligned within the region of the real normal sample; 2) reads from different regions were merged and processed to generate BAM files by using SAMtools (3).

Performance evaluation

All the CNV calls of exons predefined in simulation experiment were used as the golden standard to evaluate the ability of DeAnnCNV in detecting CNVs. For evaluation of CNV detection performance, exons with CNV (copy number ≠ 2) were treated as positives, and copy neutral (copy number = 2) exons were treated as negatives. For each sample, true positives (TP) are defined as positive exons that are correctly detected as positives by DeAnnCNV, true negatives (TN) are defined as negative exons that are correctly detected as negatives, false positives (FP) are defined as negative exons that are wrongly detected as positives, and false negatives (FN) are defined as positive exons that are wrongly detected as negatives. Three performance measurements, precision, recall and F-measure, are employed to evaluate the CNV detection performance of DeAnnCNV, which are defined as follows:

$$precision = \frac{TP}{TP + NP} \tag{7}$$

$$recall = \frac{TP}{TP + NF} \tag{8}$$

$$Fmeasure = \frac{2 \times precision \times recall}{precision + recall} \tag{9}$$

Furthermore, the real normal sample that used to generate the simulated samples was used as the common reference to call CNVs on the simulated samples.

2. Supplementary Figure

Figure S1. Screening for potential disease-causing CNVs according to the detected and annotated results provided by DeAnnCNV server.

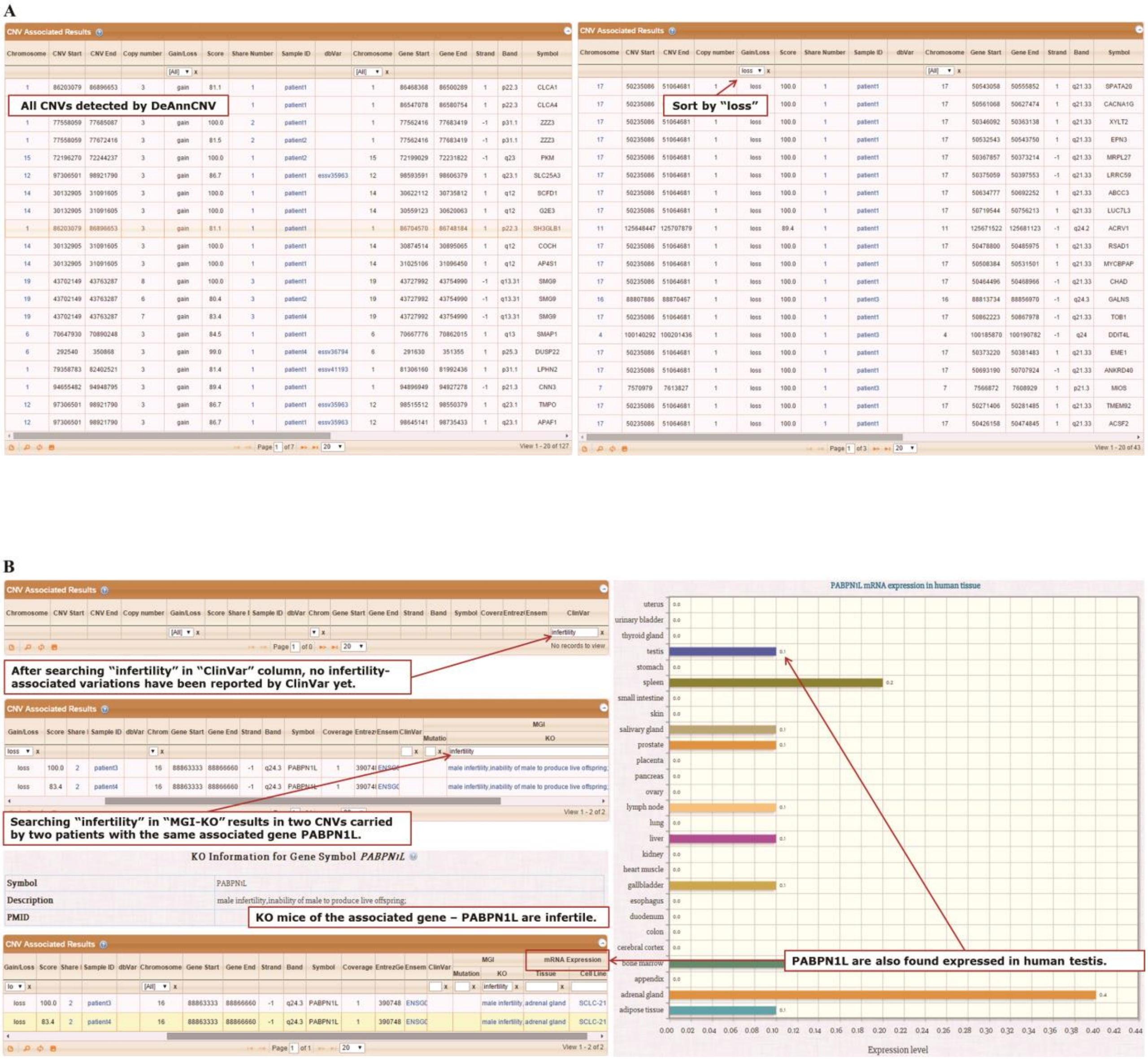

3. Supplementary Tables

Table S1. The hidden states of the HMM in DeAnnCNV.

State	Copy number	CNV type
1	0	Loss
2	1	Loss
3	2	Neutral
4	3	Gain
5	4	Gain
6	5	Gain
7	6	Gain
8	7	Gain

Table S2.Precision, recall and F-measure of DeAnnCNV for 10 simulated samples.

Samples	Measurements		
	Precision	Recall	F-measure
s1	0.99	0.98	0.99
s2	0.99	0.98	0.99
s3	0.99	0.96	0.98
s4	0.99	0.98	0.99
s5	0.99	0.99	0.99
s6	0.99	0.95	0.97
s7	0.99	0.92	0.96
s8	1	0.99	0.99
s9	0.99	0.96	0.97
s10	0.98	0.98	0.98

Table S3. Confusion matrix.

Copy number	0	1	2	3	4	5	6	7
**1**	0	7101(99%)	67	0	0	0	0	0
**3**	0	1	206	5033(96%)	0	0	0	0
**4**	0	0	25	0	850 (97%)	0	0	0
**5**	0	0	252	0	0	6956 (96%)	4	0
**6**	0	0	112	0	0	5	3284(97%)	0
**7**	0	0	284	0	0	0	5	4490(94%)
**15**	0	0	44	0	0	0	0	2119(98%)
**20**	0	0	8	0	0	0	0	602(99%)

The number of exons was counted for different copy numbers.

Table S4. Simulated CNVs for sample s1.

Region id	Chromosome	Start position	End position	Copy number
1	1	23895345	24395375	20
2	1	45570376	47070376	15
3	6	30790713	33290743	7
4	9	107420007	110920007	6
5	11	62405040	66905040	5
6	5	43574748	44074778	4
7	15	43009890	44509920	3
8	3	52529380	55029410	3
9	4	70108573	73608603	1
10	1	160817636	165317666	1

Table S5. Simulated CNVs for sample s2.

Region id	Chromosome	Start position	End position	Copy number
1	14	70581527	71081557	20
2	21	43310046	44810076	15
3	11	63790121	66290151	7
4	2	42326089	45826119	6
5	14	91124034	95624064	5
6	18	12496426	12996456	4
7	16	20241656	21741686	3
8	6	33380656	35880686	3
9	13	25332273	28832303	1
10	12	50165804	54665834	1

Table S6. Simulated CNVs for sample s3.

Region id	Chromosome	Start position	End position	Copy number
1	14	70278730	70778760	20
2	18	20000452	21500482	15
3	15	41500439	44000469	7
4	3	30004587	33504617	6
5	5	140003491	144503521	5
6	20	31122650	31622680	4
7	21	32000409	34500439	3
8	8	22221310	25721340	3
9	21	43357034	44857109	1
10	4	80012733	84512763	1

Table S7. Simulated CNVs for sample s4.

Region id	Chromosome	Start position	End position	Copy number
1	3	44374646	44874676	20
2	3	50070768	51570798	15
3	4	41759677	44259707	7
4	17	35015654	37515684	6
5	19	15236125	19736155	5
6	11	20585547	21085567	4
7	22	28526889	30026919	3
8	8	30072488	32572518	3
9	7	34690604	38190634	1
10	14	50440730	54940760	1

Table S8. Simulated CNVs for sample s5.

Region id	Chromosome	Start position	End position	Copy number
1	7	34840468	35340498	20
2	10	33582894	35082924	15
3	10	70167539	72667569	7
4	17	17865129	21365159	6
5	17	24802608	28302638	5
6	16	70128031	70628061	4
7	13	46405345	47905375	3
8	13	51907354	54407445	3
9	5	80010434	83512180	1
10	6	50848276	55348306	1

Table S9. Simulated CNVs for sample s6.

Region id	Chromosome	Start position	End position	Copy number
1	13	30093814	30593844	20
2	21	44044784	45544814	15
3	9	90875870	93375900	7
4	7	44768967	48268997	6
5	15	40022515	44522545	5
6	15	53761770	54261800	4
7	4	80079159	81579189	3
8	6	30735153	33239652	3
9	17	23115065	26615095	1
10	11	64383907	68883937	1

Table S10. Simulated CNVs for sample s7.

Region id	Chromosome	Start position	End position	Copy number
1	22	32897257	33397287	20
2	18	20302781	21802711	15
3	15	40249134	42749164	7
4	21	42702542	46202572	6
5	16	21180027	25680057	5
6	16	70217593	70717623	4
7	4	84599855	86099885	3
8	14	60573480	63073510	3
9	9	90279775	93779805	1
10	1	63562373	68062403	1

Table S11. Simulated CNVs for sample s8.

Region id	Chromosome	Start position	End position	Copy number
1	22	30880177	31380207	20
2	22	37028735	38528765	15
3	4	84601245	87101275	7
4	1	60103373	63613403	6
5	17	20347743	24847773	5
6	6	90405043	91905073	4
7	10	77488338	77988368	3
8	11	20579188	23079218	3
9	13	44442114	47942144	1
10	8	30770905	35270935	1

Table S12. Simulated CNVs for sample s9.

Region id	Chromosome	Start position	End position	Copy number
1	15	40505004	41005034	20
2	16	70164886	71664916	15
3	10	70081563	72581593	7
4	10	80638065	84138095	6
5	7	44805290	49305320	5
6	19	15091187	15591217	4
7	8	70701956	72201986	3
8	2	42135893	44635923	3
9	4	41772345	45272375	1
10	4	45762097	50262127	1

Table S13. Simulated CNVs for sample s10.

Region id	Chromosome	Start position	End position	Copy number
1	22	20371886	20871916	20
2	1	31199071	32699101	15
3	1	35422562	37922592	7
4	9	21005862	24505892	6
5	15	41981618	46481648	5
6	6	80777171	81277201	4
7	19	15082288	16582318	3
8	2	53751087	56251117	3
9	2	60840703	64340733	1
10	18	31993653	36493683	1

Reference

1. Dempster, A.P., Laird, N.M. and Rubin, D.B. (1977) Maximum likelihood from incomplete data via the EM algorithm. Journal of the royal statistical society. Series B (methodological), 1-38.
2. Rabiner, L. (1989) A tutorial on hidden Markov models and selected applications in speech recognition. Proceedings of the IEEE, 77, 257-286.
3. Li, H., Handsaker, B., Wysoker, A., Fennell, T., Ruan, J., Homer, N., Marth, G., Abecasis, G. and Durbin, R. (2009) The sequence alignment/map format and SAMtools. Bioinformatics, 25, 2078-2079.
